# Social Prescribing for Individuals Living with Mental Illness in an Australian Community Setting: A Pilot Study

**DOI:** 10.1007/s10597-020-00631-6

**Published:** 2020-05-13

**Authors:** Christina Aggar, Tamsin Thomas, Christopher Gordon, Jacqueline Bloomfield, James Baker

**Affiliations:** 1 School of Health and Human Sciences, Southern Cross University, Gold Coast campus, room B7.15, Southern Cross Drive, Bilinga, QLD 4225 Australia; 2 Susan Wakil School of Nursing and Midwifery, Faculty of Medicine and Health, The University of Sydney, Sydney, NSW 2006 Australia; 3 Primary and Community Care Services, Unit 7, 1 Central Avenue, Thornleigh, NSW 2120 Australia

**Keywords:** Behavioural health, Case management, Community healthcare

## Abstract

Social prescribing, also known as “community referral”, is a means of referring individuals living in the community to existing local non-clinical health, welfare, and social support services. International evidence demonstrates that social prescribing improves biopsychosocial quality of life, and burden on health services. Australia’s first social prescribing pilot program for individuals with mental illness (mood and psychotic spectrum disorders) was implemented in Sydney in 2016/2017; this study evaluates that program. Participants included 13 adults who were assessed at baseline and six-month follow-up. Outcomes included self-perceived quality of life, welfare needs, health status, loneliness, social participation, and economic participation. Results indicate significant improvements in quality of life and health status. This pilot program demonstrates that social prescribing may improve participant outcomes. It fits well within Australian health policy and funding models which focus on bolstering community care, and may be scalable, particularly in geographically isolated communities.

## Introduction

Mental illness impacts individuals and the wider community and places a considerable financial burden on the health system (AIHW 2016). Mental illness is affected by biological, psychological, and social factors, and treatments targeting a range of these factors, including the wider determinants of health, are more likely to be effective (WHO 2005). Social prescribing programs address these biopsychosocial factors via care coordination and linkage where individuals with mental illness are referred to local community-based social care services and structured social activities (Knapp et al. 2012). These services can be public, private, or volunteer services, and address a broad range of needs across biopsychosocial domains including physical health (medication management, disease-specific groups, diet, exercise), psychological health (support groups, counselling), welfare (food, housing, employment), and social support (group activities, befriending services).

Social prescribing for mental illness may have a role to play in the Australian contact as, in Australia, mental illness is experienced by approximately 45% of people aged 16–85 years during their lifetime, and 20% of the population experience mental illness each year (ABS 2008). Mental illness accounts for 12% of the total burden of disease and 23.6% of the non-fatal burden of disease (AIHW 2016). Between 2014 and 2015, Australian mental health service spending was approximately $8.5 billion, and the estimated total cost of the burden was $98.9 billion, 6% of the gross domestic product (AIHW 2016; RANZCP 2016).

Hospital emergency departments (EDs) are frequently the first point of contact for individuals seeking mental health services, accounting for 3.4% of ED visits in public hospitals (AIHW 2016). However, this is expensive and generally unnecessary; in 2014–2015 the majority (60.7%) of mental health ED visits were resolved without the need for admission. In addition to EDs, mental illness is frequently treated in general practice (GP). In Australia in 2014–2015, an estimated 12.7% of GP visits were due to mental health-related issues, primarily depression, including 2.9 million government-supported services (AIHW 2016; Britt et al. 2015). This rate is increasing by approximately 6.1% annually since 2010, placing pressure on GP resources (Britt et al. 2015). The most common course of treatment is medication which does not target the range of biopsychosocial factors that contribute to mental illness and does not produce the most effective long-term outcomes (Britt et al. 2015; Davey 2008).

The limitations and expense of EDs and GPs treating mental illness is recognised in Australian health policy which aims to increase the services provided in the community (AHMAC 2013). The strategy is recovery-oriented and promotes evidence-based services that provide biopsychosocial support. Additionally, the strategy aims to embrace new models of community care (AHMAC 2013). An approach which is well suited to the current Australian mental illness service strategy and addresses the current population needs is social prescribing. Social prescribing has a focus on community development, community capacity-building and creating social capital, and has the potential to address the individual and societal impacts of mental illness by improving Quality of Life (QoL) and decreasing burden (Knapp et al. 2012).

Social prescribing programs can be broadly focussed, for example the Expert Patients Program, where participants attended self-management groups to improve self-efficacy, confidence, and QoL (Rogers et al. 2008). Group education included exercise, meal planning, symptom management, relaxation techniques, and partnering and communicating with physicians. Participants demonstrated improved perceived physical health, QoL, health self-efficacy, and reduced healthcare utilisation and costs (Rogers et al. 2008). Similar broadly-focussed programs can involve case-management and referral to a range of community-based services, including parent groups, disease-specific support groups, alcohol and drug support groups, and welfare advice (Grant et al. 2000; Kimberlee et al. 2014; Windle et al. 2009). Further, more targeted initiatives involve specific group activities such as bush walking and horticulture, community group exercise, arts and crafts, and time-banking (Bragg et al. 2013; Burgess 2014; Potter 2015; Stirrat 2014).

In terms of physical health, social prescribing programs demonstrate efficacy in improving overall self-reported health status, increased physical activity, and improved energy and fatigue (Bragg et al. 2013; Druss et al. 2010; Kimberlee et al. 2014; Lorig et al. 2001). Social prescribing can also improve psychological health, including QoL and wellbeing, depression and anxiety, mood disturbance and anger, and health self-efficacy (Bragg et al. 2013; Lorig et al. 2001; Potter 2015; Rogers et al. 2008). Social prescribing studies demonstrate efficacy in improving social participation including community participation and improved social support (Bragg et al. 2013; Burgess 2014; Potter 2015), and economic participation by increasing rates of employment and mean household income (Burgess 2014; Kimberlee et al. 2014).

In terms of burden on the health system, social prescribing can decrease the number of hospital admissions, outpatient visits, mean length of hospital stay, number of GP visits, allied health appointments and prescription medication usage (Kimberlee et al. 2014; Loughren et al. 2014; Rogers et al. 2008; Windle et al. 2009). The financial impact of these savings is also demonstrated (Kimberlee et al. 2014; Windle et al. 2009). A meta-analysis of social prescribing studies by Knapp et al. (2012) concluded that relatively low-cost investments in community capital-building initiatives can result in sizeable public savings.

Social prescribing programs have not been widely implemented, nor evaluated, in Australia. A not-for-profit non-government organisation that delivers community-focussed health care, developed and implemented Australia’s first social prescribing program for mental illness, the Plus Social program. Participants diagnosed with mental illness living in the community under the care of a GP, were assessed by a social worker, and referred to appropriate locally-based health and welfare services and social activities. The program aimed to improve QoL and wellbeing, health self-efficacy, and social and economic participation.

This paper reports on the evaluation of the Plus Social program. The primary objective of this study was to evaluate whether the pilot program improved QoL, and social and economic participation. The research questions informing the evaluation were: (1) can a Plus Social program improve QoL including psychological wellbeing, physical health, welfare needs, loneliness and self-efficacy; and (2) can a Plus Social program improve social and economic participation?

## Methods

A pre-post analysis of de-identified data was undertaken.

### Design

Evaluation of the pilot program used an exploratory, quantitative, longitudinal design. Data were collected pre-intervention (baseline) and at six months (follow-up).

## Participants

Participants were required to be aged 18–65 years, living in the community in the Sydney Local Health District, and diagnosed with serious mental illness likely to last 6 months or longer. Serious mental illness is one that is severe and persistent, with complex needs - a “mental, behavioural, or emotional disorder resulting in serious functional impairment, which substantially interferes with or limits one or more major life activities” (NIMH 2019).

Potential participants were excluded if they were receiving current acute inpatient treatment or had a significant cognitive impairment.

Participants were patients who self-presented to their GP and were referred into the Plus Social program if the GP determined they met the inclusion criteria, had unmet biopsychosocial needs, and were likely to benefit from inclusion in the program. Participants were offered enrolment in the study by their GP.

### Procedures

Participants were assessed in their home by a mental health social worker; social workers completed all tools by reading questions aloud to participants, with the exception of the welfare needs and support assessment (Camberwell Assessment of Need Short Appraisal Schedule; Slade et al. 1999) which the social workers completed based on their own assessment of participant needs. Baseline data collection comprised assessment of participant wellbeing and welfare needs to inform referral to services. Link workers discussed potential services with participants and provided service information and referrals. Services included the Connecting Care Chronic Disease Management Program, the NSW Health Housing and Accommodation Support Initiative, the Personal Helpers and Mentors service, and the Acute Post-Acute Care ‘Hospital in the Home’ service (ACI 2014; DSS 2013; Muir 2007; NSLHD 2015).

All participants attended a weekly arts and crafts group (2–3 h for 10 weeks); groups were led by a practicing artist/instructor and co-facilitated by a mental health social worker who maintained communication with participants throughout the program, and provided additional supports and adjustments pre- and post-activities, for example arrangements for transportation. All participants received ongoing support and review both from their GP and social worker throughout the program.

### Materials

#### Quality of Life

The World Health Organisation QoL tool WHOQoL measured overall QoL (1 item) and health satisfaction (1 item), and four domains including physical health, psychological health, social relationships, and environment. Higher scores indicated greater perceived QoL and health satisfaction (WHOQoL Group 1998).

#### Welfare Needs and Support

The Camberwell Assessment of Need Short Appraisal Schedule CANSAS measured health and welfare needs including accommodation, food, financial, physical, psychological, and social needs. Needs rated as “Met”, “Unmet”, or “No Problem” (Slade et al. 1999).

#### Health Status and Health Self-efficacy

The EuroQol Health Thermometer EQ5D measured perceived health state on a visual analogue scale from 0 *‘worst imaginable health state’* to 100 *‘best imaginable health state’*. One item assessed health self-efficacy: *“I am confident in my ability to take action when my health status changes”*, rated 1 (strongly disagree) to 5 (strongly agree) (Herdman et al. 2011).

#### Psychosocial Distress

The Kessler Psychological Distress Scale K10 measured global psychosocial distress including agitation, fatigue, and depression in the past four weeks. Higher scores indicate greater distress (AMHOCN 2005; Kessler et al. 2002).

#### Loneliness and Social Participation

The UCLA 3-item Loneliness Scale measured how frequently participants feel left out, isolated, or lacking companionship. Higher scores indicate greater loneliness. One item assessed frequency of social participation in the previous two weeks, rated 1 (never) to 4 (frequently) (Hughes et al. 2004).

#### Economic Participation

One item measured participation in paid employment (yes/no) in the previous 2 weeks.

#### Hospital Admissions

One item measured number of hospital admissions (for any reason) in the previous 6 months. Note that due to small sample sizes and low incidence, this measure is not reported in the findings.

### Data Analysis

Data were entered, cleaned, checked, and analysed in SPSS 22 (IBM 2013). Data were checked for normality and analysed per distribution characteristics. Within and between-group analysis was conducted, and significant differences considered when *p* < 0.05. Within-group differences across time (changes in participant wellbeing, and social and economic participation) were analysed using Dependent *t*-tests and Wilcoxon signed-rank tests for non-parametric data. Between-group differences (variables related to participant retention and attrition) were analysed using independent *t*-tests, Mann Whitney U tests for non-parametric data, and Chi-Squared tests for categorical data. As the program was a pilot, the study was exploratory in nature and the probability of Type I errors arising due to multiple comparisons was not considered a major concern. As such, we did not do any corrections for multiple comparisons. For the same reasons we did not use intention-to-treat to account for attrition. We wanted to examine all comparisons to determine if statistical significance demonstrated important clinically meaningful associations.

Ethical approval was granted by The Southern Cross University HREC (2016/560). Author CA has received research grants from Primary and Community Care Services, and author JBa is the CEO of Primary and Community Care Services, NSW, Australia. All authors certify responsibility for the manuscript.

## Results

### Participant Characteristics

Twenty-four participants commenced the program and 11 were lost to follow-up (54% retention). The final sample included 13 participants (*n* = 9 female, 69%) with an average age of 45 years (*SD* = 15).For participants who completed the program, the most common primary diagnoses were depression (*n* = 3) and anxiety (*n* = 3), and most common problems were anxiety (*n* = 2), stress (*n* = 2), and sleep disturbance (*n* = 2). In terms of participants who withdrew from the program, the most common primary diagnosis was bipolar disorder (*n* = 4); all of these participants withdrew (100% attrition). The most common severe disorder for participants who withdrew from the study was anxiety (*n* = 4). There were no significant demographic differences (all *p* > 0.05) between participants who did or did not withdraw.

### Biopsychosocial Wellbeing

Participant’s scores for all questionnaires with testing of change from baseline to follow-up outlines below. Means for baseline (*M*_*B*_) and follow-up (*M*_*F*_) are presented with degrees of freedom following in parentheses.

#### Quality of Life (WHO-QoL)

Self-report overall health satisfaction (*M*_*B*_ = 2.4(1.00), *M*_*F*_ = 3.3(0.97), *t*(10) = 3.194, *p* < 0.05), physical QoL (*M*_*B*_ = 11.6(1.78), *M*_*F*_ = 13(3.620), *t*(10) = 2.451, *p* < 0.05), and psychological QoL (*M*_*B*_ = 10.3(2.45), *M*_*F*_ = 12.4(2.96), *t*(10) = 2.909, *p* < 0.05) improved significantly from pre- to post-intervention. However, overall QoL (*M*_*B*_ = 3.3(0.45), *M*_*F*_ = 3.5(0.90), *t*(10) = 1.305, *p* = 0.221), social relationships (*M*_*B*_ = 12.3(3.63), *M*_*F*_ = 12.9(4.24), *t*(10) = 1.041, *p* = 0.322), and environment QoL (*M*_*B*_ = 14.4(2.67), *M*_*F*_ = 14.8(3.21), *t*(10) = 1.44, *p* = 0.18) did not improve significantly.

#### Welfare Needs and Support (CANSAS)

Both met (*M*_*B*_ = 5.0(2.48), *M*_*F*_ = 5.5(3.53), *t*(10) = 0.268, *p* = 0.794) and unmet (*M*_*B*_ = 3.8(1.21), *M*_*F*_ = 5.5(5.57), *t*(10) = 1.025, *p* = 0.33) health and welfare needs (e.g. accommodation, food, financial, physical, psychological and social needs) tended to increase, however there were no significant changes.

#### Health Status (EQ5D) and Health Self-efficacy

Self-report health status improved significantly from pre- to post-intervention (*M*_*B*_ = 59.1(18.68), *M*_*F*_ = 71.7(14.82), *t*(9) = 2.964, *p* < 0.05). Health self-efficacy was moderately high over the course of the study, and did not change significantly from pre- to post-intervention (*M*_*B*_ = 3.6(1.26), *M*_*F*_ = 3.7(0.78), *t*(11) = 0, *p* = 1).

#### Psychological Distress (K10)

Overall psychological wellbeing was moderate-high (Andrews and Slade 2001) over the course of the study, and a despite a trend towards improving psychological wellbeing there were no significant differences in distress from pre- to post-intervention (*M*_*B*_ = 25.3(9.38), *M*_*F*_ = 22.8(8.23), *t*(11) = 1.145, *p* = 0.277).

#### Loneliness (UCLA 3-Item Loneliness Scale) and Social Participation

There were no significant differences in self-rated loneliness from pre- to post-intervention (*M*_*B*_ = 6.4(2.29), *M*_*F*_ = 6.1(2.17), *t*(10) = 0.412, *p* = 0.689), however scores trended downwards indicting participants experienced less loneliness over the course of the study. There were no significant differences in social participation from pre- to post-intervention (*M*_*B*_ = 2.7(1.32), *M*_*F*_ = 2.9(0.94), *t*(10) = 0.232, *p* = 0.821).

#### Economic Participation

Participant economic participation was low across the study. At baseline three participants (23%) reported economic participation in the previous 2 weeks, and this decreased to one participant (8%) at follow-up; Wilcoxon Signed-Rank *Z* = 1, *p* = 0.317.

## Discussion

This pilot study aimed to evaluate the Plus Social program, a social prescribing program for mental illness, implemented in Sydney, Australia. The primary study objective was to evaluate whether the program improved QoL, and social and economic participation. Participants who completed the program experienced significant improvement in physical and psychological QoL, health satisfaction and self-perceived health status. There were no significant differences in social participation and self-rated loneliness, although scores indicated participants experienced less loneliness over the course of the study, and economic participation remained low.

Patients who reported improvement in self-perceived health status experience increased independence and improvement in their perceived ability to self-managed their health/mental health conditions (Bragg et al. 2013). Previous findings reported in the grey literature suggest that patients accessing similar programs have higher levels of satisfaction with the support they receive and feel better supported to manage their condition (Dayson and Bashir 2014).

Health self-efficacy was moderately high over the study duration and did not improve significantly from pre- to post-intervention. This finding may be due to the nature of the sample, whereby participants were required to independently present to a GP and consent to participation in the program, and thus may have had generally high health self-efficacy. Additionally, it may be due to the focus of the program. For example, the Expert Patients Program primarily targeted health self-efficacy; the goal of the intervention was to “reinforce the value and salience of people’s pre-existing self-care activities, rather than initiating alternative behavioural changes” (Rogers et al. 2008, p. iii). Post-intervention, participants reported significantly improved self-efficacy, significantly better QoL, and lower service utilisation (e.g. fewer GP practitioner visits) (Rogers et al. 2008). Whilst the current study also improved QoL and resources utilisation, the mechanism of change was focussed on behaviours, and thus possibly not mediated by self-efficacy, resulting in a non-significant change.

Social prescribing has a focus on community development, community capacity-building and creating social capital, and has the potential to address the individual and societal impacts of mental illness by improving QoL and decreasing the burden of mental illness (Knapp et al. 2012). International social prescribing programs have reported direct economic health-related resource and cost benefits, reducing financial burden on the health system (Loughren et al. 2014).

In terms of scalability, social prescribing utilises existing services and is easy to implement as linkages between potential participants and their GP (point of identification/recruitment) already exist (Rogers et al. 2008). Additionally, social prescribing can target minority groups (as these support groups frequently already exist), and individuals facing disadvantage (with linkages to free, local services). Existing grey literature suggests that whilst health care professionals may be beneficial in terms of creating positive psychological and physical health gains and the ability to self-manage, volunteers could provide group facilitation to improve long term connectivity (Dayson and Bashir 2014). This pilot program demonstrated the potential efficacy of social prescribing in the Australian context. Social prescribing fits well within Australian health policy which focusses on bolstering community care; could easily be included in the current Primary Health Network funding model; and may be scalable to the Australian context, including geographically isolated communities.

### Limitations

While a strength of this pilot study was the use of validated tools for data collection, there are some clear limitations. Findings are only generalisable to a Sydney Local Health District in Australia, as the small sample size was likely to impact on external validity. Further impacting sample size was a high loss to follow-up (46% drop out rate), however, there were no other significant demographic differences between participants who did or did not withdraw; no further differences between these groups were explored. It is unclear whether the low participation rates were due to the mental health of the patients, the program, or something else; for example, the most common severe disorder for participants who withdrew from the study was anxiety which may have directly impacted retention. All participants diagnosed with bipolar disorder withdrew from the study, which is consistent with previous research indicating high attrition rates in this group (Moon et al. 2012). Only two retained participants were diagnosed with a psychotic spectrum disorders; unfortunately, psychotic symptoms were not assessed which may have further elucidated this finding. Future research could consider alternative retention strategies for these groups; in particular, qualitative interviews may elucidate strategies for retention, as may psychoeducation regarding adherence (Moon et al. 2012). We were also unable to establish the number and diagnosis of those who declined to participate in the study which represents a selection bias and may have skewed the results in favour of better functioning individuals. Despite the limitations of the pilot study in terms of power and generalizability, it is encouraging that participants did demonstrate improvements in QoL, however future studies with larger samples and improved selection techniques are needed to validate these findings.

An additional limitation was a lack of long-term follow-up of participants, which is not uncommon in this type of study, and should be considered in future research (Bickerdike et al. 2017). For example, at twelve months following the Wellspring Healthy Living Centre's Wellbeing program, 29% of beneficiaries obtained employment. Additionally, participants reported significantly fewer GP visits, significantly shorter GP visits, and a decline of 14% in medication prescriptions (Kimberlee et al. 2014). Therefore, longer follow-up periods could allow further insight into the social and financial outcomes of social prescribing programs.

## Implications for Behavioral Health

The results from this pilot program are promising; there were significant improvements in a range of biopsychosocial health and economic outcomes, suggesting that social prescribing would be an appropriate intervention in Australian settings. Use of volunteers for group facilitation to improve long-term social connectivity in future research is also recommended.

## Data Availability

The datasets supporting the conclusions of this article are available from the corresponding author upon request.

## Abbreviations

CANSAS: Camberwell Assessment of Need Short Appraisal Schedule
ED: Emergency Department
EQ5D: EuroQol Health Thermometer
GP: General Practitioner
K10: Kessler Psychological Distress Scale
QoL: Quality of Life
UCLA: University of California, Los Angeles
WHO-QoL: World Health Organisation Quality of Life
